# 4-Hexylresorcinol: pharmacologic chaperone and its application for wound healing

**DOI:** 10.1186/s40902-022-00334-w

**Published:** 2022-02-01

**Authors:** Seong-Gon Kim

**Affiliations:** Department of Oral and Maxillofacial Surgery, College of Dentistry, Gangneung-Wonju National University, Jibyun-dong, Gangneung, Gangwondo 28644 Republic of Korea

## Abstract

4-Hexylresorcinol (4HR) is amphiphilic organic chemical and auto-regulator for micro-organism. As 4HR administration induces the stress on the endoplasmic reticulum, 4HR changes protein folding. The application of 4HR inhibits NF-κB signal pathway and TNF-α production. In addition, 4HR administration increases VEGF, TGF-β1, and calcification associated proteins. As a consequence, 4HR administration increases angiogenesis and bone formation in wounded area. Strong anti-inflammatory reaction and capillary regeneration in diabetic model demonstrate that 4HR can be applied on many types of surgical wound.

## Background

Uneventful wound healing is a dream for every surgeon. Considering surgical procedure accompanies with iatrogenic trauma in the patient, full recovery from operation is an important issue in surgery. However, wound healing is influenced by many factors such as surgeon’s skill, systemic condition of patients, medications, and supplementary procedures [1]. Many of them are under the control by operator. However, there is a limitation in correcting systemic condition of patients. Particularly, the systemic diseases associated with aging influence badly on wound healing. Recovery after surgery is also highly different between young and geriatric patients. In case of third molar extraction, elderly patients have higher rate of complications [2]. In spite of rapid progress in medical technology, aging and death are inconvincible topics until now. Therefore, the development of accelerated wound healing technology is neither the level of curing any type of systemic disease nor the level of young again. It is aimed to change local environment temporarily as favorable for wound healing. Pharmacologic chaperone is a small organic chemical and can change protein folding as desirable direction [3]. The application of pharmacologic chaperone can be considered for challenging cases of unhealed wound caused by some systemic diseases.

**Fig. 1:**
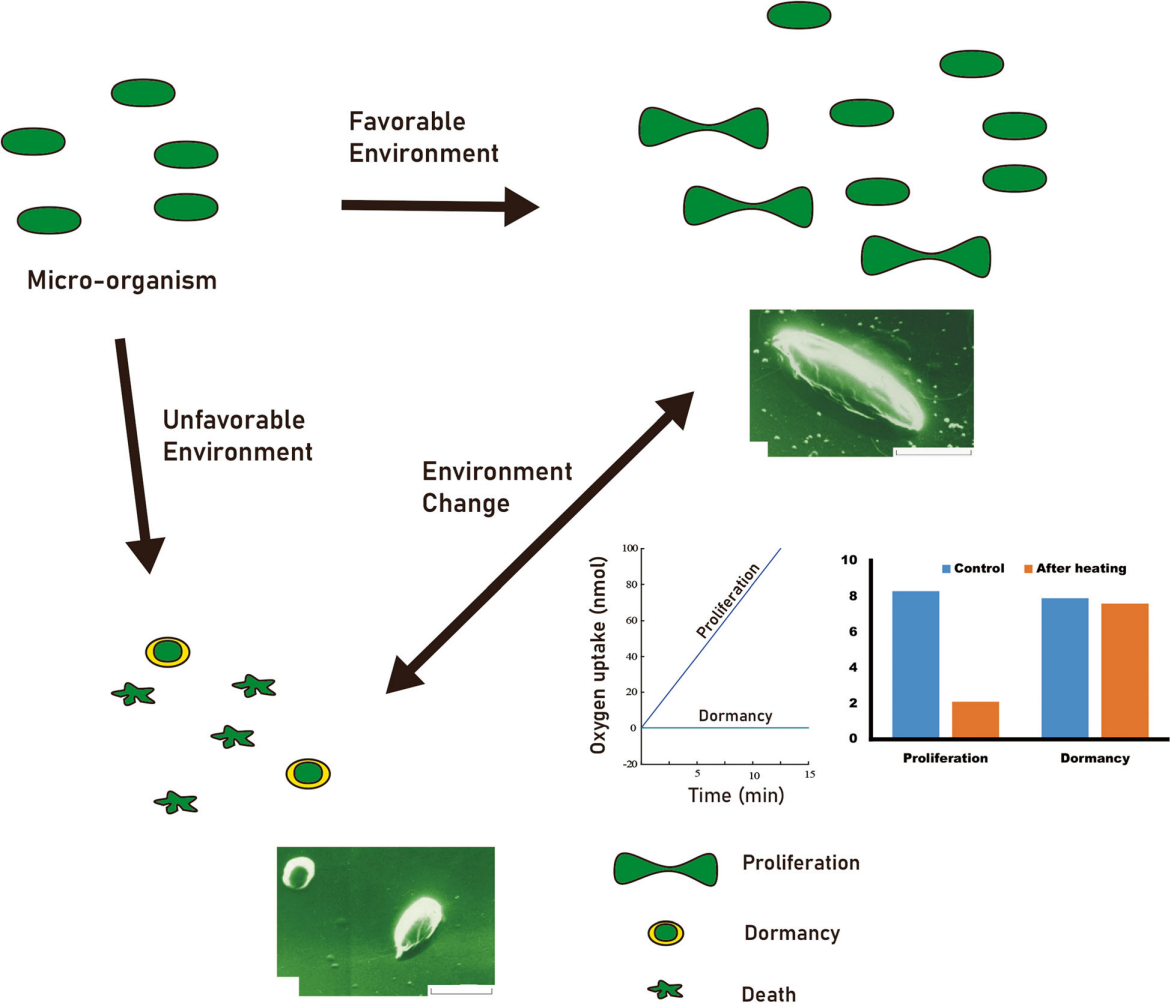
The metabolism of micro-organism is dependent on outer environment. If the environment is unfavorable, many micro-organisms will stop their proliferation and undergo dormancy. The micro-organism in the dormancy does not use oxygen for its metabolism. In addition, it can survive after heating unlike the micro-organism under proliferation. The morphology of micro-organism is also different between proliferating one and dormant one (image clip and data were provided by Dr. Andrew)

Micro-organism increases active proliferation under favorable environment. However, its proliferation is suppressed under unfavorable environments such as shortage of nutrients, anti-septic application, and extreme temperature [4]. Most proliferating micro-organism will be died under unfavorable environment. Few in the dormancy can survive in harsh environment and will be revertant at environmental change [4]. Chaperone produced by micro-organism is a kind of alarming signal to neighbors for helping successful transformation to dormancy [5]. Chaperone can bind to protein and changes protein conformation [3, 5]. Chaperone-protein complex generally stabilizes protein conformation and this complex is more resistant to environmental stress [4]. Any micro-organism failed to respond this signal will be dead (Fig. 1). In spite of favorable environment, if chaperone is given to active proliferating micro-organism, this signal will be considered as the same that is given in real emergency [6]. Accordingly, micro-organism will stop its proliferation and undergo dormancy or die due to energy imbalance [6, 7].

4-Hexylresorcinol (4HR) is a synthetic organic material, and its structure is based on resorcinolic lipids [8]. Resorcinolic lipids have been produced in micro-organism and plants [8]. Its production is increased under environmental stress such as dry climate, high/low temperature, and attack by other lives [6, 7]. In case of micro-organism, the administration of resorcinolic lipid induces dormancy like state [6, 9]. The micro-organism in the dormancy state resist environmental stress and result in species preservation [4]. This action of resorcinolic lipid is defined as chemical chaperone [8]. Micro-organism under favorable environment increase their proliferation [4]. The application of 4HR to active proliferating bacteria induces vegetative state and suppresses bacterial growth via suppressing respiration [6, 7]. Because of this function, 4HR has been used for anti-septic or anti-parasitic agent [10].

4HR has 6 carbon alkyl group, and this alkyl group is highly hydrophobic [8]. Many enzymes have hydrophobic pocket as their substrate binding site. Therefore, 4HR may inhibit many types of enzymes. Tyrosinase is involved in transformation of tyrosine to melanin [11]. 4HR is a strong inhibitor of tyrosinase [11]. When fresh apple or shrimp is harvested, they will change their color as black. Thus, 4HR treatment of these foods can prevent melanosis after harvesting. As the absorption rate of 4HR from gastro-intestinal tract is low, 4HR has been used as safe food additives [12]. Folding change of proteins after bindings of 4HR increases the stress of endoplasmic reticulum (ER) [13]. Interestingly, ER stress may induce cellular differentiation or apoptosis according to cell types [14]. There may be some proteins in the extra-cellular spaces which can bind to 4HR. These proteins may behave like decoy proteins for 4HR. These interactions would decrease any adverse systemic effect of 4HR when it is administered locally. However, systemic administration of 4HR may deliver small amount of 4HR to the target organ. In addition, it may increase the incidence of off-target effect. Gaining insights into cellular mechanism of 4HR will shed light on the role of 4HR in wound healing and lead to novel therapeutic strategies for challenging wound.

## Main text

### 4HR and transforming growth factor-β1 (TGF-β1)

TGF-β1 is a master cytokine which can regulate all cellular process in wound healing [15]. The main function of TGF-β1 in tissue is keeping homeostasis following trauma or infection [15]. Dysregulation of TGF-β1 in wound healing can be chronic inflammation or excessive fibrosis. TGF-β1 controls proliferation and survival of T cells in peripheral tissue [16]. TGF-β1 can suppress self-destructing immune attack by peripheral T cells [17]. Accordingly, TGF-β1 knockout mice show autoimmune disease and die due to serious organ inflammation [18]. High level of TGF-β1 in wounded area recruit many kinds of immune cells including macrophages [19]. In later stage of wound healing, macrophages secrete TGF-β1 for effective repair [20]. TGF-β1 produced by macrophages invite vascular endothelial cells, epithelial cells, and fibroblasts for restoring tissue integrity [21]. As TGF-β1 is overexpressed in both chronic inflammation and undesirable fibrosis [22, 23], simply application of TGF-β1 may not be enough for uneventful wound healing. TGF-β1 should be expressed as controlled manner.

The administration of 4HR to many types of cells increases the expression level of TGF-β1 [24, 25]. In case of human umbilical vein endothelial cell (HUVEC), 4HR increases the expression level of vascular endothelial growth factor (VEGF) via TGF-β1-mediated pathway [26]. The suppression of TGF-β1 by siRNA or antibody decreases the expression level of VEGF after 4HR administration [25]. Classically, hypoxia inducible factor (HIF) is known to strong activator of VEGF [27]. However, 4HR induced VEGF is HIF-independent, but TGF-β1-dependent [26]. When tissue is wounded, vascular network will be damaged. In this case, local oxygen supply will not be enough to meet the demand of tissue. To avoid excessive blood loss, the vessel should be constricted at initial stage. However, local oxygen tension askes much more oxygen and nutrient supply. Accordingly, HIF is increased its expression level and VEGF expression will be accompanied [27, 28]. The expression of VEGF at this stage is mainly responsible for vasodilatation and increased vascular permeability [27]. Many immune cells and nutrients will be supplied to wounded area via this mechanism. When there is a transition from inflammatory phase to healing phase, angiogenesis is an essential step. The increased level of TGF-β1 at this stage will contribute on capillary regeneration and fibrosis. Therefore, VEGF expression mediated by TGF-β1 at this stage will increase capillary regeneration, not vasodilatation [25]. In this point of view, surgeon may need TGF-β1-mediated VEGF expression, not HIF-mediated VEGF expression for uneventful and rapid wound healing. In other opinion, HIF-2α-mediated VEGF expression is important in capillary regeneration [28]. Interestingly, 4HR administration does induce neither HIF-1α nor HIF-2α [26].

**Fig. 2:**
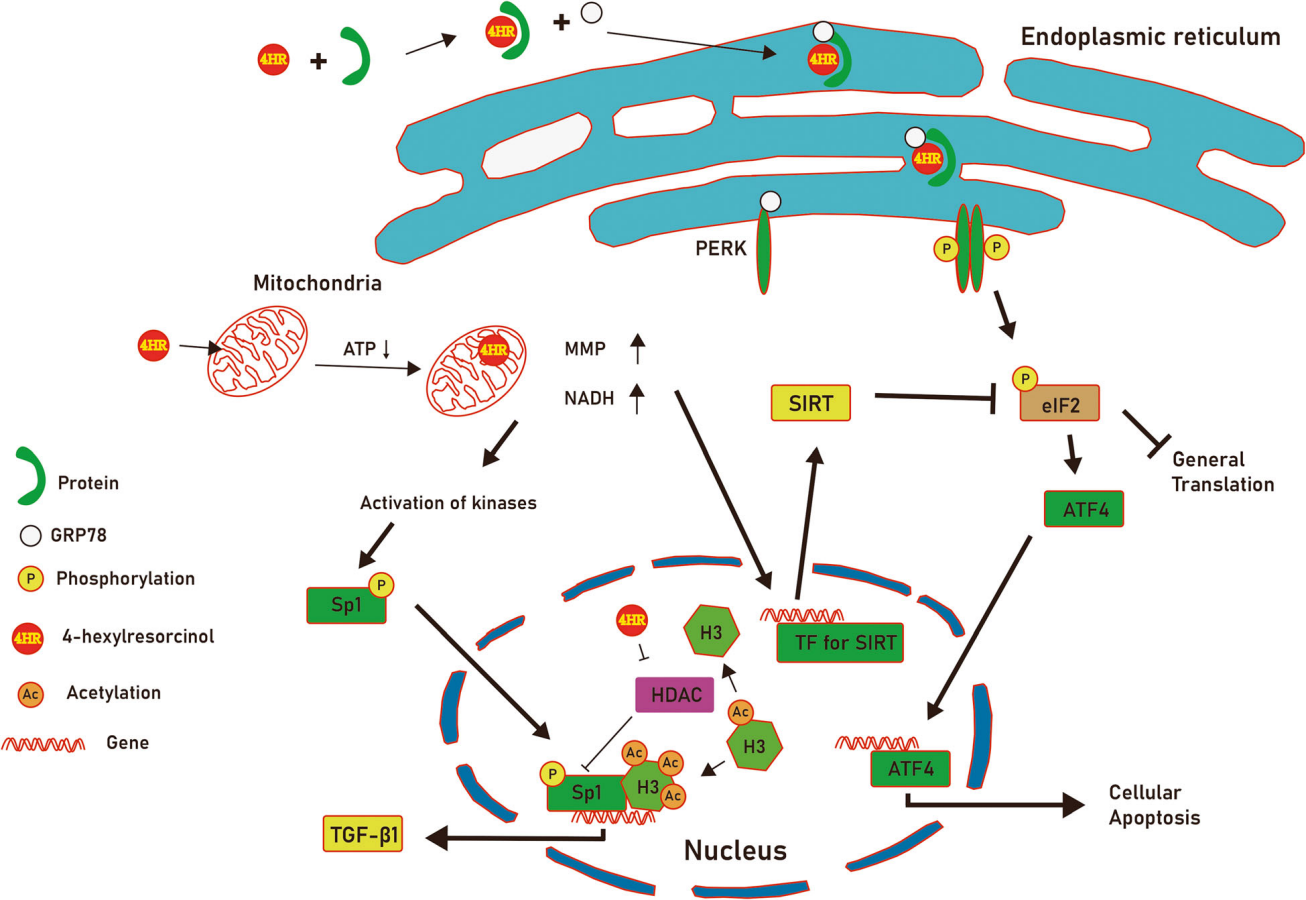
The mitochondrial and endoplasmic reticulum (ER) stress induced by 4-hexylresorcinol (4HR). The protein bound to 4HR changes its conformation. It is recognized by GRP78 and GRP78 tagged protein is transported to ER. It increases phosphorylation of PERK and eIF2, sequentially. It suppresses general gene translation and induces ATF4 induced cellular apoptosis. By the way, 4HR suppresses mitochondrial respiration and ATP synthesis. Mitochondrial membrane potential (MMP) and nicotinamide adenine dinucleotide hydrate (NADH) are increased sirtuin (SIRT) expression and its activity. SIRT can suppress eIF2 activity and this alleviates ER-induced apoptotic stress. Mitochondrial stress activates many kinases and Sp1 phosphorylation. Decreased histone deacetylase activity by 4HR administration increases acetylation of histone 3 (H3). Sp1 phosphorylation and hyper-acetylation of H3 increases transforming growth factor-β1 (TGF-β1) transcription activity (reproduced from author’s own work [13])

The mechanism of 4HR-induced TGF-β1 expression has been partly unveiled recently. As 4HR can bind to many proteins via hydrophobic interaction, its binding may result in conformational change of target proteins. It is evident from the finding that 4HR increases ER stress [13]. ER stress increases cellular differentiation or apoptosis [14]. The expression of TGF-β1 can be increased by ER stress (Fig. 2). The application of ER stress inhibitor can alleviate 4HR-induced TGF-β1 expression [13].

### 4HR and histone deacetylase (HDAC)

**Fig. 3:**
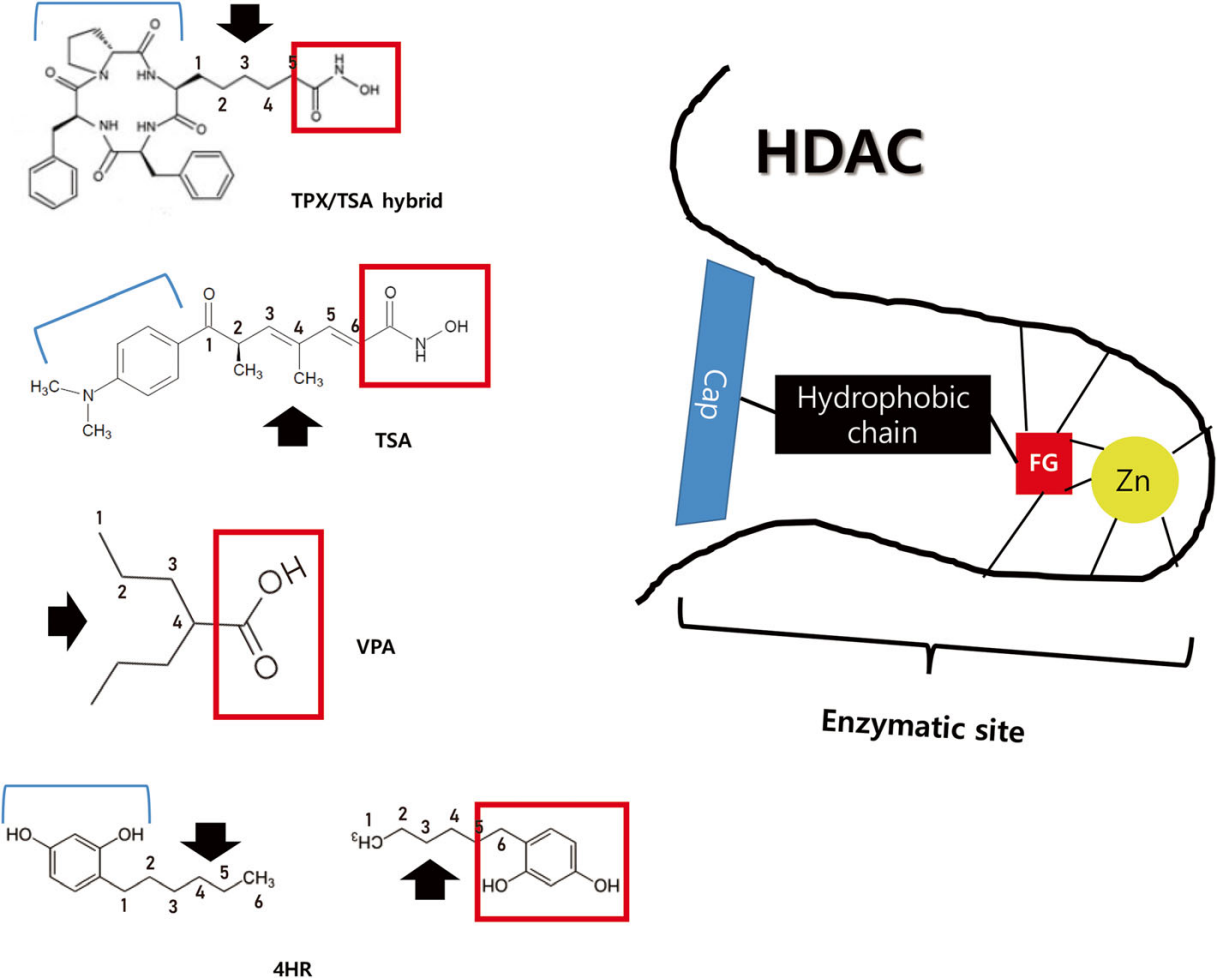
Structures of known histone deacetylase (HDAC) inhibitor and 4-hexylresorcinol (4HR). Most HDAC inhibitors have 4–6 carbon length alkyl group. This alkyl group is a hydrophobic chain and corresponds to hydrophobic domain of HDAC. As zinc ion is required for HDAC activation, some HDAC inhibitors have functional group (FG). To block any introduction of substrate into HDAC enzymatic site cap domain (Cap) can be found in some HDAC inhibitor. Interestingly, hydroxyl-benzene in 4HR can be behaved like FG or Cap (modified from author’s own work [32])

HDAC is an enzyme to remove the acetyl group from histone [29]. As the acetyl group can be added during post-translational modification, HDAC can influence on the function of many proteins [30]. As DNA is bound to histone, HDAC can influence on gene expression [29]. Many types of diseases have shown elevated activity of HDAC [29, 31]. Therefore, HDAC inhibitor can have a therapeutic benefit on many types of diseases. Until now, many kinds of HDAC inhibitor have been introduced (Fig. 3). As HDAC has several classes, HDAC inhibitor also decreases certain class of HDAC. Until now, most HDAC inhibitors are the inhibitor for class I or II HDAC.

**Fig. 4:**
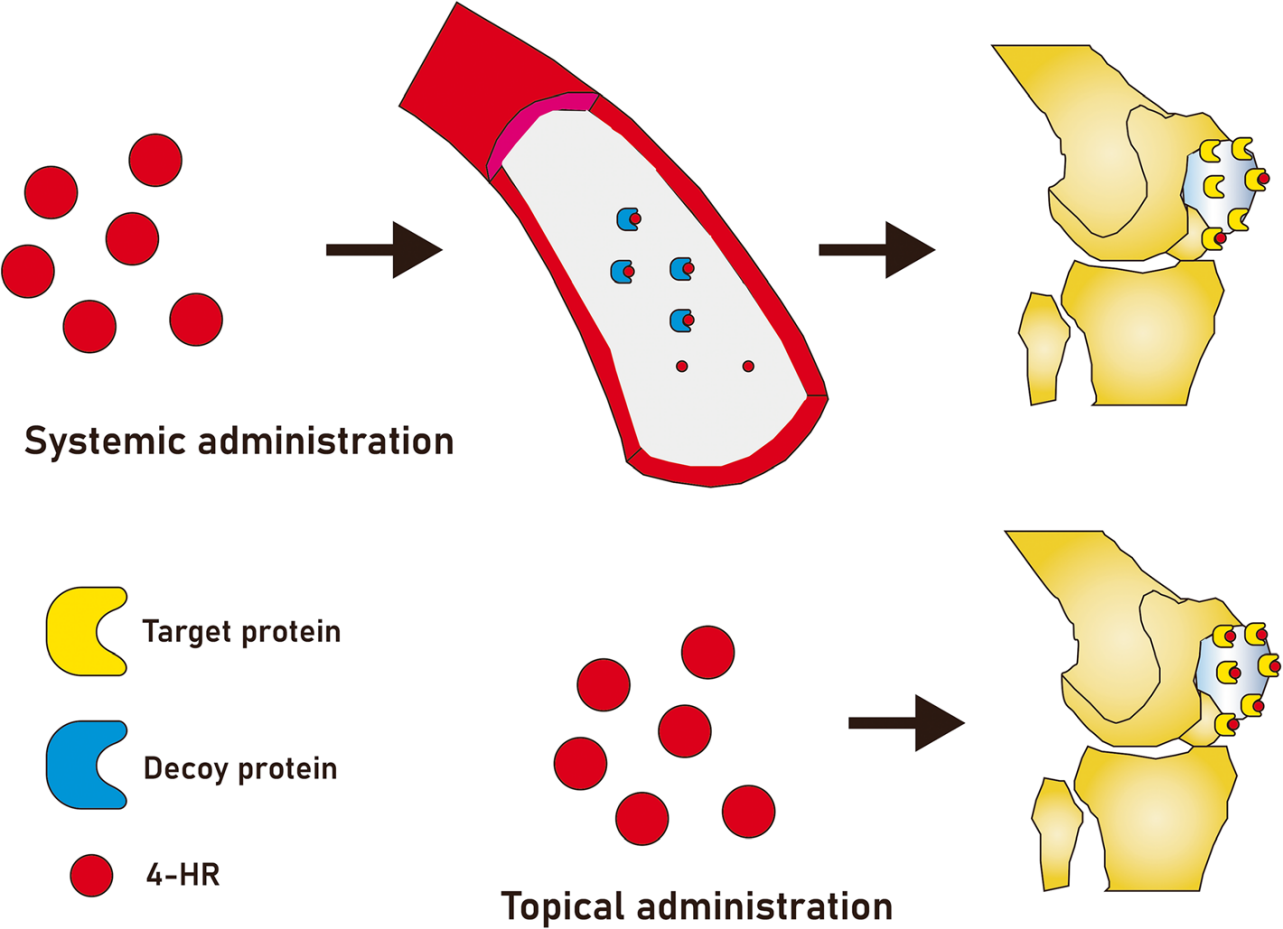
Comparison between systemic administration and topical application. Systemic administration of 4HR can be done via per os, subcutaneous, or intravenous route. In any case, administered 4HR will introduce into vessel. The decoy protein for 4HR can be found in anywhere. When 4HR is bound to decoy protein, the amount of delivery to the target organ will be decreased. If wound healing is aimed, topical delivery of 4HR will be much better. If target disease is osteoporosis, 4HR should be administered systemically. In this case, smart tool for drug delivery may be required to avoid unnecessary off-target effect

Trichostatin-A (TSA) is a representative HDAC inhibitor [33]. TSA is hydroxamic acid and suberoylanilide hydroxamic acid (SAHA) is included in the same class [34]. Benzamides, electrophilic ketones, cyclic tetrapeptides, and short-chain fatty acid are another examples of HDAC inhibitor [35]. The molecular structure of TSA is composed of cap, hydrophobic chain, and zinc chelating domain  (Fig. 3). Based on these functional units, modification of TSA can produce many derivatives of TSA and they have various activity against HDAC [36]. Valproic acid is a short-chain fatty acid and has been used as anti-epileptic drug [37]. Though valproic acid does not have cap structure, it has both hydrophobic chain and zinc chelating domain [36]. Accordingly, valproic acid is also HDAC inhibitor. 4HR has cap and hydrophobic chain, but does not have zinc chelating domain (Fig. 4). However, the administration of 4HR decreases class I HDAC activity [32]. 4HR can be considered as a short-chain fatty acid [8,8].

Class IIa HDAC is important in muscle remodeling. HDAC4 accumulation in the nucleus is frequently found in denervated muscle [38]. Sarcopenia is associated with aging, and HDAC4 is elevated its expression in the aging related atrophic muscle [39]. HDAC4 is also associated with neurogenic muscle atrophy [40]. Though there was no report on 4HR and age-associated muscle atrophy, 4HR administration decreased the expression level of HDAC4 in both HUVECs [32] and Saos-2 cells [41]. The administration of HDAC inhibitor accelerates muscle regeneration [42]. Blocking Class I HDAC delays the progression of Duchenne muscular dystrophy [43]. The administration of valproic acid enhances motor nerve regeneration and motor function [37].

The application of HDAC inhibitor to transformed cells usually induces cellular apoptosis and inhibits tumor growth [44, 45]. The administration of 4HR has anti-cancer effects on oral cancer cells [46, 47]. The application of HDAC inhibitor usually decreases inflammation via suppressing the expression level of tumor necrosis factor-α (TNF-α) [48]. The administration of SAHA to peripheral blood mononuclear cells decreases TNF-α expression [49]. Thus, the application of SAHA has a protective effect on joint showing arthritis [50]. Interestingly, the administration of 4HR also decreases TNF-α expression in burns [51]. Accordingly, the application of 4HR ointment accelerates wound healing in deep burns model [51]. HDAC inhibitor can inhibit NF-κB signaling pathway [48]. HDAC3 activates NF-κB signaling pathway and increases the expression level of many pro-inflammatory genes [52]. HDAC inhibitor may suppress HDAC3 and results in hyperacetylation of signal transducer and activator of transcription 1 (STAT1) [53]. Hyperacetylated STAT1 has strong anti-inflammatory effect [54].

When cell receives oxidative stress such as hydrogen peroxide application, 4HR can protect cells via elevating anti-oxidant enzymes as  resveratrol does [26, 55, 56]. This may be a chaperone-like protection of 4HR. However, the administration of 4HR to proliferating cell induces mitochondrial stress via suppressing mitochondrial respiration activity [13]. To ameliorate 4HR-induced mitochondrial stress, the expression level of sirtuin 6 (SIRT6) and sirtuin activity are increased after 4HR administration [13]. Though 4HR-induced ER and mitochondrial stress increases the apoptotic pressure on cells, compensatory mechanism for cellular survival such as anti-oxidant and SIRT activity is also increased after 4HR administration [13, 56]. However, these responses can be different to cellular type or environment.

SIRT is class III HDAC [30]. Unlike class I or II HDAC, SIRT is NAD-dependent deacetylase [30]. SIRT1 has therapeutic effect on type 2 diabetes [57] and Alzheimer’s disease [58]. In case of yeast, overexpression of SIRT2 gene increases its lifespan up to 30% [59]. SIRTs are usually insensitive to TSA [60]. SIRT1 decreases fat accumulation in adipose tissue [61]. SIRT1 increases glucose-stimulated insulin secretion from pancreatic β cells and decreases glucose intolerance [62]. SIRT1 overexpression inhibits NF-κB pathway [63]. SIRT1 also inhibits oxidative stress via increasing cyclooxygenase-2 expression [64]. SIRT1 is important for survival in the calorie restriction environment [65]. SIRT6-deficient animal shows premature senescence [66]. Until now, SIRT activating molecules are polyphenolic compounds. Among them, resveratrol has been widely studied [67]. However, resveratrol has not been widely used as SIRT activator because of its off-target effect [68] and difficulty in achieving SIRT1 activating serum concentration [69]. Interestingly, 4HR administration increases SIRT activity and NAD+ level [70]. However, off-target effect of 4HR should be screened before its usage as SIRT activator. The subcutaneous injection of 12.8 mg/kg 4HR weekly for 12 weeks in a 4-week-old male rat results in decreased mandibular size and serum testosterone level [41]. As the testis is metabolic active organ with high number of cellular mitosis, the energy demand from the mitochondria may be most higher organ in the body. Thus, the suppression of mitochondrial energy production by 4HR administration may suppress testis activity. Actually, 4HR has spermicidal action [71]. However, 4HR administration may not block the synthetic pathway of testosterone. It is evident by logical deduction from the finding that the production of testosterone from the salivary gland in the same animal model is increased by 12.8 mg/kg 4HR weekly injection (unpublished data).

### Clinical application of 4HR as wound healer

4HR has been used as antiseptcis [10] and food additives [11]. Considering its poor absorption rate through gastro-intestinal tract, high dosage of 4HR had been administered to the patients having tropic enteritis [72]. Some young patients showed hypersensitive, but the nature of hypersensitivity was poorly defined [72]. HDAC inhibitor may increase calcification. The administration of 4HR increases TGF-β1, bone morphogenic protein-2 (BMP-2), runx2, osteocalcin, and osteopontin in osteoblast-like cell [41] and dental pulp cells [56]. Thus, 4HR administration per os may be considered for the patients having osteoporosis. Recently, China team demonstrated 4HR administration per os has a therapeutic effect on osteoporosis in the animal model [73].

4HR has been studied as anti-cancer drug [46, 47]. According to toxicological studies, regular 4HR administration per os showed preventive effect on spontaneous carcinogenesis in the lab animals [74]. However, this anticancer drug-like effect may be harmful for uneventful wound healing because most anticancer drugs suppress cellular proliferation. Actually, high dosage of 4HR decreases serum testosterone level in growing male rats [41] and shows spermicidal effect in rabbits and monkeys [71]. In spite of this flaw as wound healer, 4HR administration has many benefits on healing process. As mentioned above, 4HR increases osteogenesis-associated proteins and calcification [41, 56]. Therefore, 4HR incorporated bone graft would have some benefits. 4HR incorporated silk fibroin graft inhibits foreign body giant cell formation and increases new bone formation [75]. Unlike other anti-cancer drug, 4HR increases VEGF expression level and angiogenesis [76, 77]. Interestingly, 4HR-induced angiogenesis is HIF independent [26]. Thus, 4HR administration increases capillary regeneration regardless of local oxygen tension [25]. This may prevent proliferation of immature endothelial cells which is commonly found in chronic inflammation.

4HR is also strong suppressor for TNF-α and IL-1β [56, 78]. Both cytokines are responsible for active inflammation. High level of TNF-α in deep burns results in shock and multiple organ dysfunction syndrome [79]. Topical application of 4HR ointment accelerates wound healing in deep burns via suppressing the expression level of TNF-α [51]. Chaperone-like behavior of 4HR will be helpful for cellular survival in wounded area. 4HR can suppress ROS-associated cellular damage through its anti-oxidant effect [26, 56]. SIRT activity is increased by 4HR administration [13, 70], and it may also contribute on cellular survival in wounded area. 4HR-mediated TGF-β1 expression will increase fibrosis in the connective tissue which is an essential step for epithelial wound healing. Reduced oxygen tension with mitosis suppression by 4HR administration may suppress keloid formation, too. However, this is just logic deduction and should be proved by experiments.

Diabetes is a metabolic disorder. The patients having diabetes show the problem in wound healing. High level of serum glucose level suppresses phagocytosis and immunoglobulin-mediated opsonization [80]. In addition, high level of serum glucose level increases ROS production [81]. As a consequence, chronic inflammation with poor capillary regeneration is frequent in diabetic wound [82]. 4HR is strong M2 macrophage polarizing agent [26, 83]. The administration of 4HR will increase M2 types of macrophages [26]. In addition, high level of ROS and TNF-α in the diabetic wound will be decreased by 4HR administration [25]. Chaperone-like behavior of 4HR will suppress mitochondria-based cellular metabolism and increase SIRT activity [13, 70]. This will improve survival rate of residual cells in wounded area. Actually, 4HR topical administration accelerates wound healing in skin burns and oral ulcer of diabetic animals [25, 51]. Interestingly, 4HR administration decreases blood sugar level in diabetic animals, but detailed mechanism is unclear (data not shown). 4HR administration improves survival of diabetic rats after intra-oral burns, but the difference among groups is not significant (*P*>0.05).

According to recent study, topical application of 4HR is much better therapeutic effect than systemic administration in arthritis model (data not shown). Topical application of 4HR on joint showed improved bone mineral density and decreased joint inflammation, but there was no significant difference in 4HR systemic administration group compared to the untreated control group. The reason of this different effect is unclear. Considering that 4HR is a chaperone, its target proteins are many. Thus, 4HR may not reach to the therapeutic level in case of systemic administration because many decoy proteins bind to 4HR and there will be very small free 4HR in the serum (Fig. 4). As topical application can be done on the target organ, its diffusion distance is very short and off-target effect will be minimal. Topical application of 4HR accelerates epithelialization in the defect after partial maxillectomy [1]. Topical application of 4HR on exposed dental pulp shows rapid healing and wound closure [56]. 4HR incorporated silk fibroin plug shows similar level of bone regeneration to collagen plug [84].

Other applications of 4HR in dentistry may be the application on orthodontics. 4HR administration increases tooth movement rate in ovariectomized animal model [85]. 4HR administration also reduces tooth root resorption after excessive orthodontic force application [86]. Incisor eruption rate in the rat is increased by 4HR administration [87]. Xenograft induced host immune response can be suppressed by 4HR pretreatment [88]. SARS-CoV-2 antibody-mediated serious inflammation results in bad consequence after COVID-19 infection [89]. Interestingly, 4HR shows some therapeutic effect on COVID-19 infection [90]. Detailed mechanism is unclear. Virus is undergoing dormancy frequently until replication. If SARS-CoV-2 viral protein utilizes its hydrophobic domain as tool for binding to the receptor of target cells, 4HR may bind viral protein and neutralize via inducing dormancy and blocking the binding to target protein. In addition, its anti-inflammatory action via suppressing TNF-α in host cells will be also helpful for avoiding serious inflammation after COVID-19 infection. However, respiratory disease is out of scope and this hypothesis should be clarified by further experiment.

## Conclusion

Pharmacologic chaperone has been shown therapeutic effect on various diseases associated with protein folding disorder. 4HR induces dormancy in micro-organism and increases ER stress. Accordingly, 4HR should be considered as pharmacologic chaperone. The application of 4HR suppresses inflammation via inhibition of NF-κB pathway and TNF-α. The application of 4HR increases capillary regeneration and calcification. Accordingly, 4HR can be used for accelerating intra-oral wound healing.

## Data Availability

Data sharing is not applicable to this article since no dataset was generated or analyzed during the current study.

## Abbreviations

4HR: 4-Hexylresorcinol
COVID-19: Coronavirus disease of 2019
HDAC: Histone deacetylase
HIF: Hypoxia inducible factor
HUVEC: Human umbilical vein endothelial cell
IL-1β: Interleukin-1beta
NAD: Nicotinamide adenine dinucleotide
NF-κB: Nuclear factor-kappaB
ROS: Reactive oxygen species
SAHA: Suberoylanilide hydroxamic acid
SARS-CoV-2: Severe acute respiratory syndrome coronavirus 2
SIRT: Sirtuin
STAT1: Signal transducer and activator of transcription 1
TGF-β1: Transforming growth factor-beta 1
TNF-α: Tumor necrosis factor-alpha
TSA: Tichostatin-A
VEGF: Vascular endothelial growth factor
